# Prevalence and risk factors of retro-styloid lymph node metastasis in oropharyngeal carcinoma

**DOI:** 10.1080/07853890.2022.2031270

**Published:** 2022-01-31

**Authors:** Ryo Toya, Tetsuo Saito, Yoshiyuki Fukugawa, Tomohiko Matsuyama, Tadashi Matsumoto, Shinya Shiraishi, Daizo Murakami, Yorihisa Orita, Toshinori Hirai, Natsuo Oya

**Affiliations:** a Department of Radiation Oncology, Faculty of Life Sciences, Kumamoto University, Kumamoto, Japan; b Department of Diagnostic Radiology, Faculty of Life Sciences, Kumamoto University, Kumamoto, Japan; c Department of Otolaryngology-Head and Neck Surgery, Faculty of Life Sciences, Kumamoto University, Kumamoto, Japan

**Keywords:** Head and neck cancer, radiotherapy, oropharyngeal carcinoma, lymph node metastasis, retro-styloid lymph node, positron emission tomography, clinical target volume

## Abstract

**Background:**

Supporting data defining the selection criteria of level VIIb for inclusion in the target volume in radiotherapy (RT) planning are insufficient. We evaluated the prevalence of level VIIb retro-styloid lymph node metastasis (RSLNM) and associated risk factors in patients with oropharyngeal carcinoma (OPC).

**Materials and methods:**

We retrospectively reviewed pre-treatment [^18^F]-fluoro-2-deoxy-d-glucose–positron emission tomography/computed tomography (CT) along with contrast-enhanced thin slice CT and magnetic resonance (MR) images of 137 patients pathologically confirmed as having OPC who underwent RT. The location of lymph nodes (LNs) was confirmed on the planning CT images. Fisher’s exact test and logistic regression analyses were made to determine the risk factors of RSLNM.

**Results:**

RSLNM was confirmed in 18 (13%) patients. All RSLNMs were located within level VIIb on the planning CT images. No patients exhibited LNM in contralateral level VIIb. Furthermore, no patients with negative or single ipsilateral cervical LNM had RSLNM. Fisher’s exact test revealed that smoking status (*p*=.027), multiple ipsilateral cervical LNM (*p*=.045) and LN ≥15 mm in the upper limit of ipsilateral level II (*p*<.001) were significantly associated with RSLNM. Logistic regression analyses revealed that the presence of LNs ≥15 mm in upper limit of ipsilateral level II was significantly associated with RSLNM (odds ratio: 977.297; 95% confidence interval: 57.629–16573.308; *p*<.001).

**Conclusions:**

RSLNM is relatively common in patients with OPC with a prevalence rate of approximately 10%. The prevalence of RSLNM in patients with negative or single ipsilateral cervical LNM and contralateral RSLNM is extremely low; therefore, level VIIb can be excluded from the target volume in such patients. LN ≥15 mm in the upper limit of ipsilateral level II is a risk factor for RSLNM. Ipsilateral level VIIb should be included in the target volume for patients with this risk factor.

**KEY MESSAGE**
- Retro-styloid lymph node metastasis (RSLNM) prevalence is ∼10% in oropharyngeal carcinoma.
- Lymph node ≥15 mm in ipsilateral level II upper limit is a risk factor for RSLNM.

## Introduction

Radiotherapy (RT) with or without chemotherapy is one of the main treatment modalities for oropharyngeal cancer (OPC) [1]. With the increased use of high-precision RT techniques, such as intensity-modulated radiotherapy (IMRT) and volumetric modulated arc therapy, which permit highly conformal dose distributions, the optimal selection and delineation of RT target volume have grown to be increasingly important [2,3]. Recommendations for selecting lymph node (LN) levels for computed tomography (CT)-based RT planning were proposed two decades ago, specifically for levels I–VI and retropharyngeal LN (RPLN), mainly based on a review of the prevalence of LN metastasis (LNM) as assessed using neck dissection specimens [4].

Retro-styloid LNs (RSLN) were first mentioned in consensus guidelines published by Grégoire et al. [5]. Currently, RSLN is defined as LN located in the retro-styloid space, labelled as level VIIb. Level VIIb is the area extending from the skull base to the caudal edge of the lateral process of C1 (upper limit of level II) and delineated by the medial edge of the internal carotid artery medially, by the styloid process and the deep parotid lobe laterally, by the C1 vertebral body and the base of skull posteriorly, and by the posterior edge of the pre-styloid para-pharyngeal space anteriorly [6]. Like RPLN, which is currently defined as located in level VIIa, RSLN metastasis (RSLNM) is difficult to diagnose by physical examination or ultrasound. Furthermore, pathological data for RSLNM are limited because a neck dissection does not extend beyond the posterior belly of the digastric muscle [4]. Radiographic modalities such as CT, magnetic resonance (MR) imaging and [^18^F]-fluoro-2-deoxy-d-glucose (FDG)–positron emission tomography (PET) are essential for the assessment of RSLNM. The assessment of the prevalence of RSLNM and associated risk factors may guide the selection criteria of level VIIb for inclusion in the target volume for high-precision RT. However, to the best of our knowledge, data clarifying the selection criteria have not yet been established. In this study, we evaluated the prevalence of RSLNM and associated risk factors in patients with OPC using imaging modalities.

## Materials and methods

### Patients

This retrospective study was approved by the Institutional Review Board of Kumamoto University Hospital (no. 2281). Between May 2011 and December 2020, 173 patients with pathologically confirmed OPC were treated with RT at our hospital. Of these, 157 patients underwent pre-treatment contrast-enhanced MR imaging within 4 weeks and FDG-PET/CT imaging within 6 weeks before RT planning CT scan in our hospital. Twenty patients were excluded because of surgery; and/or RT before imaging; and/or coexisting lung cancer, oesophageal cancer and/or head and neck cancer at other subsites. After exclusions were made, the final study population consisted of 137 patients. Prior informed consent was obtained from all patients for the treatment and use of their images in future studies. Clinical staging was performed by an institutional tumour board of head and neck radiation oncologists, otolaryngologists and radiologists. According to the Union for International Cancer Control (UICC) TNM staging system, 7th (*n* = 71) or 8th (*n* = 66) edition, staging was based on physical, endoscopic and ultrasound examinations with or without fine-needle aspiration cytology specimens, in addition to MR and FDG-PET/CT images.

### Assessment of pre-treatment images

All patients underwent diagnostic gadolinium-based contrast-enhanced MR using the 3T MR scanner (Philips Achieva TX; Philips Medical Systems, Best, The Netherlands). In addition to the conventional axial images consisting of T1-weighted image (WI), T2WI, as well as short tau inversion recovery with 5-mm slice intervals, fat-saturated eTHRIVE axial images with 1-mm slice intervals were obtained [7,8]. Iodine-based contrast-enhanced FDG-PET/CT images were obtained using a 3D PET/CT scanner (Gemini GXL 16; Philips Medical Systems, Best, The Netherlands). Dynamic contrast-enhanced scans were performed to obtain images with 2 mm slice intervals. FDG-PET/CT imaging protocols were as described elsewhere and at 4-mm slice intervals [9–11]. RT planning CT images with or without iodinated contrast media were acquired with 2.5 mm slice intervals using a CT scanner (LightSpeed RT; GE Medical Systems, Waukesha, WI), a pillow and a thermoplastic mask dedicated for RT [12].

Two board-certified radiation oncologists with an experience of 15 and 17 years in diagnosing and treating head and neck cancers reviewed the contrast-enhanced CT and MR images, FDG-PET and FDG-PET/CT fused images and RT planning CT images. Observers independently evaluated the images without prior knowledge regarding the clinical information of the patients, and disagreements were resolved by consensus. Radiological criteria used to define RSLNM were short-axis diameter ≥5 mm and necrosis and/or abnormal tracer uptake on FDG-PET/CT [13–15]. As diagnostic MR and FDG-PET/CT images were not acquired with the treatment position, correlations for level VIIb and other LN levels were made between the diagnostic and treatment positions. In addition to RSLNM assessment, which includes the short-axis diameter and the maximum standardized uptake value (SUV_max_), long-axis diameter of the largest LN in the upper limit of ipsilateral level II (caudal edge of the C1 lateral process) was recorded [5].

### Statistical analysis

Statistical calculations were performed using SPSS software version 26.0 (IBM, Armonk, NY). Cohen’s *Κ* analysis was performed to evaluate the inter-rater reliability of assessments by the two observers. Fisher’s exact test was performed to investigate potential risk factors of RSLNM using the following variables: smoking and p16 status, tumour site, T category, histological grade, ipsilateral cervical LNM, bilateral or contralateral cervical LNM, and long-axis diameter of the largest LN in the upper limit of ipsilateral level II. Factors with *p* values <.1 were taken forward into a logistic regression analysis. Smoking and p16 statuses are significantly related to each other [16], and p16 status is unknown in some patients; therefore, p16 status was excluded from the logistic regression analysis. Differences with *p* values <.05 were considered statistically significant.

## Results

### Patient characteristics and RSLNM prevalence

Patient characteristics and clinical N categories are summarized in Tables 1 and 2, respectively. Of the 137 patients, 114 were men, and 23 were women. Patient ages ranged from 38 to 85 years (median, 65 years). Of the 71 patients who were staged according to the 7th edition of UICC TNM staging system, 2 (3%), 5 (7%), 4 (6%), 51 (72%) and 9 (13%) patients were staged as I, II, III, VIA and VIB, respectively. Moreover, of the 66 patients who were staged according to the 8th edition, 40 patients were categorized as p16 positive status. Of these 40 patients, 20 (50%), 13 (33%) and seven (18%) patients were staged as I, II and III, respectively. Other 26 patients were categorized as p16 negative status. Of these 26 patients, 1 (4%), 3 (12%), 5 (19%), 13 (50%) and 4 (15%) patients were staged as I, II, III, IVA and IVB, respectively. The *Κ* value for inter-rater reliability of the assessments by the two observers was 0.933 (95% confidence interval (CI), 0.841–1.000; *p*<.001). Eighteen (13%) patients were diagnosed with ipsilateral RSLNM. All RSLNMs were located within level VIIb on the RT planning CT images. The median short-axis diameter and SUV_max_ of RSLNM were 11 mm (range 8–15) and 5.2 (range, 1.9–9.4), respectively. No patients had RSLNM in contralateral level VIIb.

**Table 1.** Patient and disease characteristics and results of Fisher’s exact test for risk factors of retro-styloid lymph node metastasis.

Variables	Total cohort (column %) *n* = 137	RSLN positive (column %) *n* = 18	RSLN negative (column %) *n* = 119	*p* Value
Smoking status				
Never	35 (26)	2 (11)	33 (28)	.027
Former	56 (41)	5 (28)	51 (43)	
Current	46 (34)	11 (61)	35 (29)	
p16 status				
Negative	37 (27)	6 (33)	31 (26)	.096
Positive	75 (55)	6 (33)	69 (58)	
Unknown	25 (18)	6 (33)	19 (16)	
Tumour site				
Tonsil	103 (75)	14 (78)	89 (75)	.761
Base of tongue	16 (12)	3 (17)	13 (11)	
Vallecula	6 (4)	1 (6)	5 (4)	
Post pharyngeal wall	5 (4)	0 (0)	5 (4)	
Soft palate/uvula	7 (5)	0 (0)	7 (6)	
T category				
T1	19 (14)	1 (6)	18 (15)	.138
T2	60 (44)	6 (33)	54 (45)	
T3	33 (24)	4 (22)	29 (24)	
T4	25 (18)	7 (39)	18 (15)	
Histological grade				
Well	15 (11)	3 (17)	12 (10)	.628
Moderate	87 (64)	10 (56)	77 (65)	
Poor	30 (22)	5 (28)	25 (21)	
Not graded	5 (4)	0 (0)	5 (4)	
Ipsilateral cervical LN metastasis				
No	16 (12)	0 (0)	16 (13)	.045
Single	15 (11)	0 (0)	15 (13)	
Multiple	106 (77)	18 (100)	88 (74)	
Bilateral or contralateral cervical LN metastasis				
No	100 (73)	10 (56)	90 (76)	.090
Yes	37 (27)	8 (44)	29 (24)	
Size of LN at the upper limit of ipsilateral level II				
<15 mm	117 (85)	1 (6)	116 (98)	<.001
≥15 mm	20 (15)	17 (94)	3 (3)	

RSLN: retro-styloid lymph node; LN: lymph node.

**Table 2.** Clinical N category according to the Union for International Cancer Control TNM staging system.

UICC	p16 status	*n*	N category (column %)								
			N0	N1	N2	N2a	N2b	N2c	N3	N3a	N3b
7th	NA	71	7 (10%)	4 (6%)	NA	3 (4%)	36 (51%)	18 (25%)	3 (4%)	NA	NA
8th	Positive	40	2 (5%)	25 (63%)	12 (30%)	NA	NA	NA	1 (3%)	NA	NA
	Negative	26	7 (27%)	3 (12%)	NA	0 (0%)	6 (23%)	7 (27%)	NA	1 (4%)	2 (8%)

UICC: Union for International Cancer Control; NA: not applicable.

### Risk factors of RSLNM

**Figure 1.**
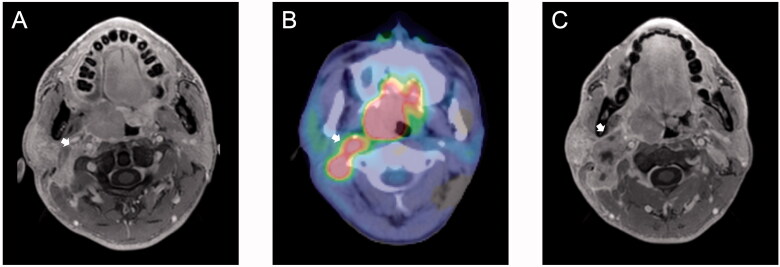
Oropharyngeal carcinoma with bilateral multiple cervical lymph nodes (LNs) and ipsilateral retro-styloid lymph node metastasis (RSLNM). (A) Contrast-enhanced fat-saturated eTHRIVE and (B) [^18^F]-fluoro-2-deoxy-d-glucose-positron emission tomography/computed tomography fused images show the presence of RSLNM (arrow; longest diameter = 14 mm, SUV_max_=4.1). (C) Contrast-enhanced fat-saturated eTHRIVE images. The longest diameter of LN in the upper limit of ipsilateral level II was 27 mm (arrow).

Fisher’s exact test revealed that smoking status was significantly associated with RSLNM (*p*=.027) (Table 1). RSLNM was more common in patients with multiple ipsilateral cervical LNM (*p*=.045). No patients with negative or single ipsilateral cervical LNM had RSLNM; therefore, this variable was excluded from the logistic regression analysis. RSLNM was also more common in patients with LN ≥15 mm in the upper limit of ipsilateral level II (*p*<.001). No significant difference was found between RSLN positive and RSLN negative groups based on p16 status, tumour site, T category, histological grade, and bilateral or contralateral cervical LNM. Table 3 summarizes results of the logistic regression analysis. The only factor significantly associated with RSLNM was LN ≥15 mm in the upper limit of ipsilateral level II (odds ratio: 977.297, 95% CI: 57.629–16573.308; *p*<.001) (Figure 1).

**Table 3.** Results of logistic regression analysis for risk factors of retro-styloid lymph node metastasis.

Variables	OR	95% CI	*p* Value
Smoking status			
Never	1		
Former	1.449	0.044–47.311	.853
Current	2.149	0.151–30.632	.573
Bilateral or contralateral cervical LN metastasis (yes)	3.949	0.340–45.884	.272
LNs ≥15 mm at the upper limit of ipsilateral level II (yes)	977.297	57.629–16573.308	<.001

OR: odds ratio; CI: confidence interval; LN: lymph node.

## Discussion

The prevalence rate of retropharyngeal LNM (RPLNM) in patients with OPC, estimated on the basis of imaging modalities, was previously reported at 10–20% [15,17,18]. In our cohort, the prevalence rate of RSLNM was 13%, which is similar to those in previous RPLNM studies. Our results suggest that RSLNM is relatively common in patients with OPC. However, including level VIIb into the target volume in all patients with OPC is unreasonable because this level is located close to the pharynx, pterygoid muscle, parotid gland and mastoid cells; irradiation to this level decreases patient’s quality of life. Therefore, the appropriate selection criteria for including level VIIb into the target volume are essential for RT planning.

In the IMRT era, the asymmetric selection of LN levels for the target volume has become popular in RT for patients with head and neck cancer. In the latest guidelines for selecting LN target volume published by Biau et al. [2], for N0–1 p16-OPC patients, level VIIb is not recommended for inclusion in the target volume. For N2–3 p16-OPC patients, ipsilateral level VIIb is recommended for inclusion in case of the bulky involvement of the upper part of level II. However, contralateral level VIIb is not recommended for target volume inclusion unless bulky contralateral involvement of the upper part of level II is present. For p16+ OPC patients, data suggesting a different selection compared with p16-OPC patients are unavailable [2]. We have been unable to retrieve sufficient supporting data for these recommendations, probably because level VIIb has only been relatively recently defined compared with levels I–VI, in which LNM is pathologically confirmed by neck dissection.

Level VIIb is the cranial continuation of level II, and it is assumed that RSLNM is closely related to the LNM status in level II. Indeed, an imaging-based classification of LN levels by Som et al. [19,20] proposed the upper limit of level II as the base of the skull. In these researchers' proposition, the retro-styloid space was included in level II [3,5]. Our results suggest that patients with LN ≥15 mm, which is the size criterion most commonly considered as LNM in level II [21], in the upper limit of ipsilateral level II are at high risk of RSLNM. This size criterion is applicable in the risk assessment of RSLNM. On the contrary, our results suggest that prevalence of RSLNM in patients with negative or single ipsilateral cervical LNM and contralateral RSLNM is extremely low. Therefore, level VIIb can be excluded from the target volume in such patients. In terms of the appropriate selection of the target volume in RT planning, our results suggest that level II and VIIb should be divided and assessed separately. Furthermore, our results strongly support the recommendations of the guidelines published by Biau et al. [2] based on the clinical data and provide additional recommendations. We believe that our suggestions contribute to the appropriate selection of LN levels for the target volume in RT planning for OPC.

Our study has some limitations. First, this study did not investigate the pathological confirmation of RSLNM, which may be unavoidable in this study design [15]. Second, this was a retrospective study involving a relatively small number of patients. The selected patients for this study were treated with RT between May 2011 and December 2020, and UICC TNM staging system had been revised in this period. Therefore, approximately half of the patients in this study were staged according to the UICC TNM staging system, 7th edition, and p16 status was unknown in some patients. There are significant differences between 7th and 8th editions in TNM staging system of OPC. In the 8th edition, different staging systems were developed according to p16 status. Furthermore, in this decade, treatment strategy for p16 positive OPC has drastically changed, and concurrent chemotherapy has been widely performed instead of surgery even for the patients with locally advanced diseases [1]. Although p16 status has no significant impact on the distribution of LN drainage [22], these factors may have influenced the findings on the prevalence of RSLNM and risk factor analysis. Further prospective evaluation of a larger patient population based on the updated treatment strategies, including p16 status, is required to confirm our suggestions on the selection criteria for target volume inclusion of level VIIb.

## Conclusions

We obtained an RSLNM prevalence of approximately 10% and observed that RSLNM is relatively common in patients with OPC. The presence of LN ≥15 mm in the upper limit of ipsilateral level II indicates a high risk of ipsilateral RSLNM. The prevalence of RSLNM in patients with negative or single ipsilateral cervical LNM and contralateral RSLNM is extremely low; therefore, level VIIb can be excluded from the target volume in such patients. These suggestions may contribute to the appropriate selection of level VIIb for inclusion in the target volume in RT planning for OPC. Further studies are required for the prospective evaluation of the robustness of our suggestions.

## Data Availability

The data supporting the findings of this study are restricted for use as they were used in this study under licence; hence, they are not publicly available. However, the data are available from the authors upon reasonable request and with permission from the Institutional Review Board of Kumamoto University Hospital.
